# Designing soft materials with interfacial instabilities in liquid films

**DOI:** 10.1038/s41467-018-06984-7

**Published:** 2018-10-26

**Authors:** J. Marthelot, E. F. Strong, P. M. Reis, P.-T. Brun

**Affiliations:** 1 Department of Chemical and Biological Engineering, Princeton University, Princeton, NJ 08540 USA; 2 Department of Mechanical Engineering, Massachusetts Institute of Technology, Cambridge, MA 02139 USA; 3 Flexible Structures Laboratory, Ecole Polytechnique Fdrale de Lausanne, 1015 Lausanne, Switzerland

## Abstract

Natural soft materials harness hierarchy and structures at all scales to build function. Adapting this paradigm to our technological needs, from mechanical, phononic and photonic metamaterials to functional surfaces prompts the development of new fabrication pathways with improved scalability, design flexibility and robustness. Here we show that the inherent periodicity of the Rayleigh–Taylor instability in thin polymeric liquid films can be harnessed to spontaneously fabricate structured materials. The fluidic instability yields pendant drops lattices, which become solid upon curing of the polymer, thereby permanently sculpting the interface of the material. We solve the inverse design problem, taming the instability, so that the structures we form can be tailored, over a range of sizes spanning over two decades. This all-in-one methodology could potentially be extended down to the scales where continuum mechanics breaks down, while remaining scalable.

## Abstract (web summary)

Interfacial instabilities can be damaging as they may lead to fabrication defects. Here the authors harness a fluid instability to their advantage to produce thin polymeric films with drop-shaped structures which have tailored geometrical properties.

## Introduction

Capillary effects are ubiquitous in inert and living matter where they dominate the dynamics of fluids at small length scales, in drops, bubbles and thin films^1^. They are central to various technological applications spanning the fields of chemical engineering, bioengineering, and material science. In their stable limit, capillary effects are routinely used as a method to straighten interfaces, e.g., in the glass-float process and in coatings on surfaces and fibers^2,3^, to functionalize surfaces^4,5^, and to deform or assemble small compliant objects^6–9^. Capillary effects also foster the fragmentation of a volume of fluid into a collection of droplets whose radii vary from tenths of nanometers to several millimeters^10–13^. These droplets serve as vehicles for biological materials^14^, for printing polymeric solutions^15,16^, or are destined to combustion^17^. However, to this day, interfacial instabilities are rarely seen as a pathway to give spatial order to materials^13,18,19^. Such structures could find applications in various fields from mechanical, phononic, and photonic metamaterials to functional surfaces^4,20–22^.

Melts and amorphous phases are universally used in manufacturing, given that they can be deformed to take a desired shape, which becomes a tangible object upon solidification. Examples include glass blowing, metal casting, plastic molding, soft-lithography, and 3D printing^14,23^. While spatial order arises from applied constraints in these engineered structures, patterns in nature often capitalize on the inherent periodicity and robustness of instabilities^24^.

Here, we show that the Rayleigh–Taylor instability in thin polymer films can be harnessed at the materials level to spontaneously shape solid structures. Specifically, thin liquid elastomeric coatings are destabilized to generate drop-shaped smooth structures with tailored geometrical properties, which are predicted by the theoretical framework we introduce. As the elastomer cures, these fluid-mediated structures yield an elastic material. We show that the robustness of the instability across length scales, and material properties are instrumental in making this passive methodology a scalable fabrication pathway.

## Results

### Tailoring the Rayleigh–Taylor instability

**Fig. 1:**
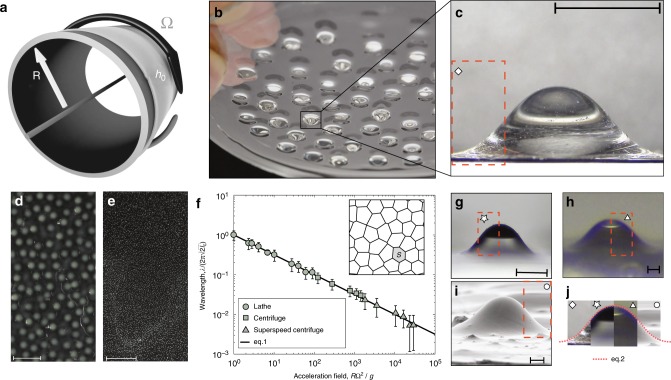
Harnessing the Rayleigh–Taylor instability at the materials scale. **a** A thin polymeric film is initially deposited onto a cylinder substrate which is then rotated so that its interface destabilizes to the Rayleigh–Taylor instability under the action of the destabilizing acceleration *a* = *R*Ω^2^. **b** Liquid polymer drops grow into a pattern that freezes as the polymer cures. **c** The resulting elastic drops may be easily peeled from the substrate. Scale bar, 5 mm. **d**, **e** Drop patterns formed with *a* = 10.2*g* and *a* = 1421*g*, respectively. Scale bars, 1 cm. **f** The lattice wavelength, *λ*, is a function of the acceleration field generating drops with size ranging from 10 mm to 50 μm. The error bars correspond to the standard deviation of measurements obtained across the sample. Inset: a typical Voronoi construction generated using the drops positions. **g**–**i** Experimental pictures and SEM images of drops obtained with an acceleration field *R*Ω^2^/*g* = 42 (**g**, scale bar 500 μm), 640 (**h**, scale bar 50 μm), 12,266 (**i**, scale bar 10 μm). **j** Photomontage combining portions of drops (**c**) and **g**–**i** compared to theory (dashed red line)

Figure 1a outlines our approach: a liquid elastomeric polymer (polydimetylsiloxane PDMS Sylgard 184, Dow Corning) is coated on the outside of a cylinder with radius *R*. The substrate is then rotated at speed Ω, either in a lathe or in a centrifuge depending on the targeted acceleration *a* = *R*Ω^2^ (see Methods). We observe the destabilization of the interface of the film into a lattice of drops. As the polymer cures, the array of liquid drops solidifies, thereby permanently sculpting this initially fluid system (Fig. 1b). The resulting structure can be used as a network, or the drops may be peeled off from the substrate and used individually (Fig. 1c), making of this method an inherently scalable fabrication pathway.

The Rayleigh–Taylor instability (RTI) in thin liquid films describes the destabilization of a fluid interface under the action of surface tension, viscous stresses and an acceleration field, usually gravity^25,26^. The drops shown in Fig. 1b, which serve as a reference case, originate from the linearly most unstable mode of the RTI in a thin film subject to the destabilizing action of gravity. The wavelength of the pattern is $\lambda _{\mathrm{M}} = 2\pi \sqrt 2 \ell _{\mathrm{c}}$, where $\ell _{\mathrm{c}}$ is the capillary length^25^. This characteristic length is obtained by balancing gravity and capillary effects so that $\ell _{\mathrm{c}} = \sqrt {\gamma /\rho g}$, where *γ* is the surface tension of the liquid, *ρ* its density and *g* is the acceleration of gravity (viscous effects only enter in setting the time scale of the instability). While gravity yields centrimetric droplets ($\ell _{\mathrm{c}} = 1.45$ mm for PDMS so that $\lambda _{\mathrm{M}} \simeq 13\,{\mathrm{mm}}$), smaller scales can be reached with the larger acceleration fields we generate via centrifugation. Typical results are shown in Fig. 1d and e, corresponding to accelerations *R*Ω^2^ = 10.2*g* and *a* = 1421*g*, respectively. In our experiments, we define the location of a drop by the coordinate of its apex. We then use this data to generate a Voronoi diagram with partitions of mean area ${\cal S}$ (inset of Fig. 1f). We define the wavelength of the lattice as $\lambda = 4/3^{1/4}\sqrt {\cal S}$. The prefactor corresponds to an ideal regular hexagonal lattice for which ${\cal S} = \sqrt {3/4} \lambda ^2$. In Fig. 1f, we show that our experiments collapse on a curve defined by:

1 $$\lambda = \lambda _{\mathrm{M}}\sqrt {\frac{g}{{R{\mathrm{\Omega }}^2}}} ,$$

where *R* is the radius of rotation and Ω the rotation speed. Equation 1 matches the classical expression of the most unstable mode of the RTI when substituting *a* = *R*Ω^2^ for *g*, thereby showing that the physics of the instability is unchanged over five decades of accelerations. We find that an acceleration ranging between *g* and 3 × 10^4^*g* yields drops with wavelength ranging from 10 mm to 50 μm, showing the robustness of the instability across length scales (see Fig. 1g–i). For accelerations larger than 10^3^*g* (triangles in Fig. 1f), we worked with silicon wafers, which were flat, so that the centrifugal forces is not uniform across the sample. This effect leads to a competition between droplets and rivulets^27^.

With the above results in hand, it is thus possible to program the instability to obtain a drop lattice with wavelength ranging over three decades, simply by modulating the magnitude of the acceleration field. The drops are remarkably similar across lengthscales as evident from Fig. 1j, where our montage is realized by rescaling each drop by $\ell = \sqrt {\gamma /\rho R\Omega ^2}$, the generalized capillary length. We anticipate that larger accelerations will produce a similar trend until other effects such as surface roughness or intermolecular forces become appreciable. We now focus on the case where *a* = *g*, and investigate thin polymeric films coated on the underside of horizontal substrates.

### From instability to order

**Fig. 2:**
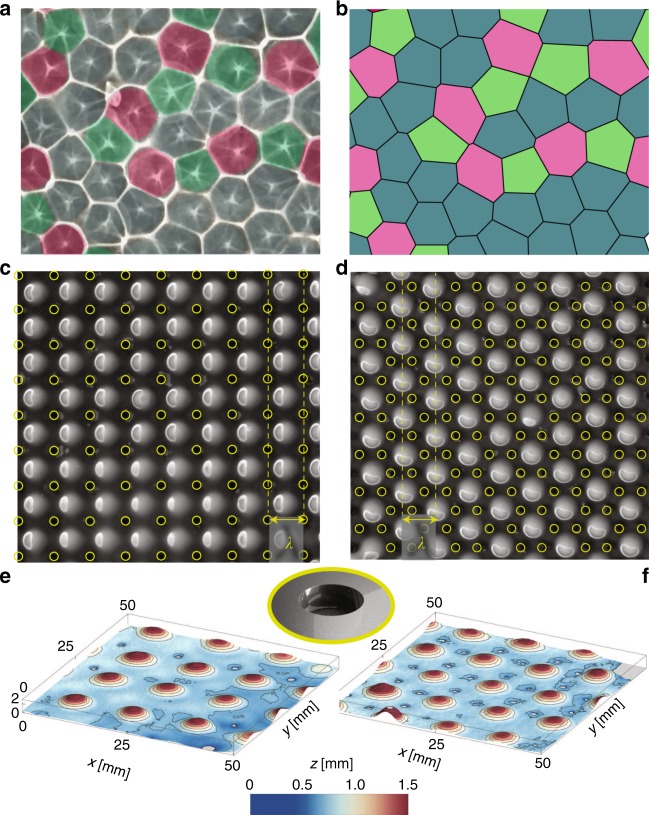
Control of the drop lattice. **a** Optical projection of the PDMS drop lattice on a flat surface. Areas are color-coded according to the type of polygon encountered. **b** Voronoi mesh of the drop lattice in **a**. Hexagons appear in blue, pentagons in green, and heptagons in pink. **c**, **d** Experimental pictures of square and hexagonal patterns forced using seeds (shown in yellow and in inset) with wavelength *λ*. **e**, **f** 3D scans of the same droplets, showing their monodispersity

We examine the regularity of the lattice that emerges spontaneously from the RTI. As the lattice cures, we are able to investigate its geometric properties with ease. In Fig. 2a, we show photographs of the caustics obtained when illuminating the translucent samples with a point source. The star-like shapes in Fig. 2a show the position of the apex of each drop, while the white segments evidence a network of flat valleys in the films separating neighboring droplets^28^. We construct the Voronoi tessellation using the drops positions, which we show in Fig. 2b. We find that the Voronoi cell boundaries coincide with the film valleys. Each drop may be seen as an isolated system, which has drained the volume initially occupying its tile. There is a majority of hexagons (blue) as well as some pentagons (green) and some heptagons (red). The lattice is thus irregular, albeit composed of tiles of approximatively the same size. Irregularities are inherent to the instability that starts from the edge of the film^25,26^ and progressively invades the sample, accumulating defects, and geometrical frustrations.

To bypass this effect and improve the monodispersity of the drops, we carefully design a layout of surface defects on the substrate. Cylindrical holes (diameter 2 mm, depth 1.6 mm), regularly spaced, are etched on the substrate (Fig. 2c, d). They are used as seed perturbations to trigger the instability simultaneously across the sample. In Fig. 2c and d, we show the results obtained with a square and a hexagonal seed layout with wavelength *λ* = *λ*_M_. The drops are not centered on the defects, but instead are located as far as possible from them, i.e., on the vertices of the dual graph of the seeds pattern. As evident from Fig. 2c and d, we are able to organize the lattice of droplets and tailor the geometry of the unit cell.

The topography of the resulting droplets pattern was digitized using a 3D laser scanner (NextEngine). Data are reported in Fig. 2e and f and color-coded such that drops apex appear in red and valleys in blue. The small cylindrical cavities evident in Fig. 2c and d mark the location of the seeds. The initial film flowed away from the seeds in a direction parallel to the sample^25^ to feed off the neighboring drops. In the classical RTI (no seeds, uniform initial coating), the growth rate of drops in a square pattern is lower than that of a hexagonal arrangement—such that square lattices are usually not observed unless forced^25^. The forcing achieved through the seeds that have been laid out in a square lattice, appears to be sufficient to promote this pattern. Note that in both the square and the hexagonal cases, we observe that the regularity of the lattice comes along with a degree of uniformity of the drops amplitude. We now aim to elucidate the underlying mechanisms that set the amplitude and the shape of these drops.

### Inverse design

**Fig. 3:**
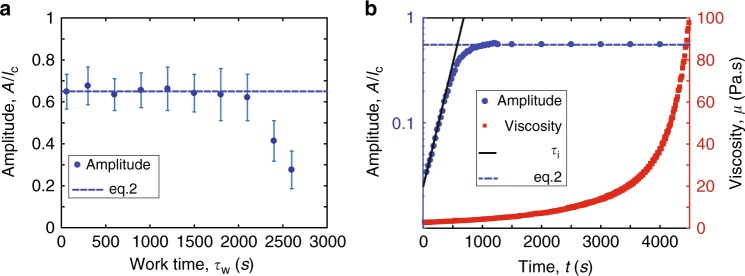
Mechanics of amplitude selection. **a** Average final amplitude of the drops normalized by the capillary length for PDMS versus the work time *τ*_w_, the time at which the plate is inverted (initial thickness of the film, *h*_0_ = 270 μm). The dashed blue line is the prediction of the final amplitude, Eq. 2. The error bars correspond to the standard deviation of the amplitude of the drops in the lattice. **b** Measured amplitude of the droplets *A*(*t*) (blue points, initial thickness of the film, *h*_0_ = 315 μm) and variation of the viscosity *μ*(*t*) (red squares). The solid black line (resp. the dashed blue line) is the prediction of the exponential growth with time scale *τ*_i_ (resp. of the final amplitude by Eq. 2)

In Fig. 3a, we report the final amplitude of drops for substrates coated with the same initial film thickness (*h*_0_ = 270 μm), while the work time *τ*_w_ at which the substrates are inverted is varied. The work time *τ*_w_ denotes the time interval between the preparation of the polymer, i.e., when curing begins, and the time at which the sample is inverted, i.e., when the gravity induced flow begins. The amplitude of the drops is averaged on 75 drops across each sample. For small values of *τ*_w_ (here *τ*_w_ < 2000 s), the final drop amplitude is independent of *τ*_w_. However, for larger values of *τ*_w_ smaller drops are obtained. We now turn to rationalize this dependance.

In our problem, the final state of the film is the result of the competition between two time scales. First, the RTI initially grows exponentially with characteristic time *τ*_i_ = 12*τ*_v_, where $\tau _{\mathrm{v}} = \mu \ell _{\mathrm{c}}^4/(\sigma h_{\mathrm{0}}^3)$ is the time scale of drainage in the thin film surrounding the drops^28^. Note that this time scale strongly depends on *h*_0_, such that small differences in *h*_0_ yield large differences in *τ*_i_. Second, the elastomer undergoes crosslinking and cures with characteristic time *τ*_c_^29^. We aim to work with physical parameters such that $\tau _{\mathrm{c}} \gg \tau _{\mathrm{i}}$. To illustrate this time scale separation, we superimpose in Fig. 3b the dynamics of the drop growth, measuring the transmittance of the PDMS coating colored with a black dye, and the time evolution of the polymer viscosity, for a constant shear rate $\dot \gamma = 0.1\,{\mathrm{s}}^{ - 1}$. We find that the predicted time scale *τ*_i_ (black line in Fig. 3b) captures the exponential growth of the instability in our experiments, which saturates in a time (700 s) significantly smaller than curing time (3500 s). Saturation is imparted by the increasing difficulty to drain fluid from the flat regions in-between the drops (as their thickness decreases). By choosing the initial thickness of the coating, we manage to operate in a regime where the time scales of the problem are separated: first the drops form and then the curing of the polymer solidifies the drops.

**Fig. 4:**
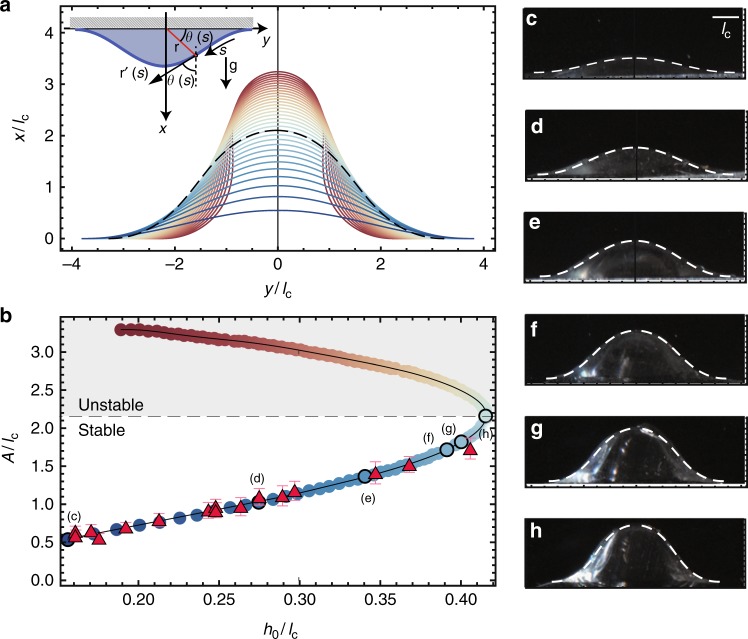
Predicting the shape of the drops. **a** Solutions of Eq.​ 2. **b** Branch of solutions in the plane $h_{\mathrm{0}}/\ell _{\mathrm{c}},A/\ell _{\mathrm{c}}$ and average experimental amplitudes (red triangles). The error bars correspond to the standard deviation of the amplitude of the drops in the lattice. **c**–**h** Experimental shapes obtained in PDMS, varying *h*_0_ compared to theoretical predictions (dashed white line)

At large times, $t \gg \tau _{\mathrm{i}}$, and before curing $t \ll \tau _{\mathrm{c}}$ the system consists of a series of drops surrounded by thin depleted films^28^. Asymptotic analysis of the equations governing this creeping flow reveals that perturbations to the drops shape relax much faster than the intervening region between drops can drain^28^. As a result, the drops are in near-equilibrium and their shape may be derived with a quasi-static approach matched asymptotically to a surrounding much thinner film. The geometry of the drops is characterized by *θ*(*s*) as defined in inset of Fig. 4a, such that the tangent to the interface is **r**′(*s*) = (cos(*θ*(*s*)), sin(*θ*(*s*))), where a prime denotes a differentiation with respect to the arc-length *s*. Given the symmetry of the problem, we search for constant-pressure solutions of

2 $$\theta \prime\prime (s) = - \ell _{\mathrm{c}}^{ - 2}{\mathrm{cos}}\theta (s) + \left( {\frac{{{\mathrm{cos}}\theta (s)}}{{y(s)}}} \right),$$

obtained by balancing gravity and surface tension effects. Integration of this equation subject to the adequate set of boundary conditions (*x*(0) = 0 and *θ*(0) = 0) is performed using a shooting algorithm leveraging on the symmetry of the problem so that the tangent to the drop is horizontal when crossing the *x* − axis (at the appex). Results obtained via this method are shown in Fig. 4a. The family of solutions are color-coded such that solutions of lesser amplitude (and volume) are marked in blue and solutions of larger amplitudes are indicated in red. All solutions are recast in Fig. 4b as a function of their dimensionless amplitude $A/\ell _{\mathrm{c}}$ and the dimensionless coating thickness $h_{\mathrm{0}}/\ell _{\mathrm{c}}$ required to form them (assuming a hexagonal arrangement of the drops to evaluate their volume, and neglecting the volume of fluid in the connecting films). The solution branch in Fig. 4b is multivalued and presents a fold separating stable solutions (small values of *A*) and unstable ones (large values of *A*, thus never observed in experiments) from regions where no static solutions exist (large values of *h*_0_). The fold-point in the curve corresponds to the drop presented as a dashed line in Fig. 4a, such that only the shapes below this curve are expected to be found in our experiments as we show next.

The amplitudes of the droplets obtained in our experiments for different initial coating thickness *h*_0_ are averaged across the sample and plotted in Fig. 4b as red triangles. To estimate the volume of PDMS trapped in the seed, we weigh the drops after peeling from the sample and recast their volume in terms of *h*_0_ using a hexagonal lattice. In Fig. 4c–h, we present photographs of cross-sectional cuts of the solidified elastic drops along with the axisymmetric solutions of the drop shapes that best fit them. We find a favorable agreement between theory and experiments. Figure 4h is the largest drop that can be obtained experimentally. Increasing the thickness of the initial film coating further leads to dripping, as no equilibrium solution exists (fold-point). The shape of the elastic interface is thus rationalized using arguments and an equation that belongs to the realm of fluid mechanics. Such a predictive model allows us to quantitatively solve the inverse problem: the targeted shape dictates the initial conditions, chosen using the explicit relation plotted in Fig. 4b. Similar arguments apply in the context of our centrifuged experiments. The targeted scale, *λ*, sets the acceleration and radius obtained reversing Eq. 1. Drop shapes are then obtained substituting the generalized capillary length $\ell = \sqrt {\gamma /\rho R\Omega ^2}$ for $\ell _{\mathrm{c}}$ in Fig. 4b. Drops with identical dimensionless volume indeed collapse on the predicted shape as shown in Fig. 1j.

### Complex elastic surfaces

**Fig. 5:**
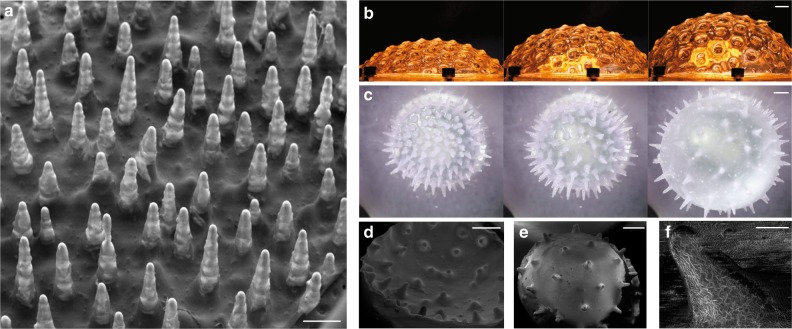
Complex elastic surfaces. **a** Hairy elastic surfaces obtained repeating our methodology 16 times with an acceleration field *R*Ω^2^/*g* = 404. Scale bar, 500 μm. **b** A hexagonal lattice of drops (*R*Ω^2^/*g* = 1) is pneumatically inflated to form a dimpled sphere. Scale bar, 10 mm. **c** Similar deformable structures are formed and actuated at the microscale (*R*Ω^2^/*g* = 404). Scale bar, 1 mm. **d**–**f** SEM images of structures (*R*Ω^2^/*g* = 632) permanently deformed on negative (**d**, scale bar, 500 μm) and positive (**e**, scale bar, 500 μm) Gaussian curvature and additional wrinkle morphology (**f**, scale bar, 20 μm) observed with an acceleration field *R*Ω^2^/*g* = 1.2 × 10^4^

At smaller scale, precisely controlling the surface defect on the substrate and the initial thickness is more challenging. We propose an alternative approach. We start with a smooth substrate, which we coat and rotate to generate an imperfect drop lattice. We then use this lattice as a substrate, which is coated and rotated again at the same speed. Each time, the previously obtained lattice serves to force the instability. After a few generations, we find that the shapes that we obtain converge towards the same aspect ratio. In Fig. 5a, we show the hairy elastic structures obtained after 16 generations of coating at an acceleration *R*Ω^2^/*g* = 404.

We then take advantage of the elastic properties of our materials and show that those structures can be dynamically actuated into a variety of convex and concave shapes. Under pressure loading, a hexagonal array of droplets with regular amplitude bulges to form a dimpled sphere (Fig. 5b and Supplementary Movie 1). This deformation is reversible owing to the constitutive properties of the elastomer. Similar deformations are obtained for hairy surfaces (Fig. 5c and Supplementary Movie 2) at an intermediate scale. At microscopic scale, two-tier controlled structures are demonstrated on surfaces permanently deformed with positive and negative Gaussian curvature and imaged with SEM (Fig. 5d, e). An additional wrinkle morphology at the surface of the structure is observed for larger values of the acceleration (*R*Ω^2^/*g* = 1.2 × 10^4^) with the multiple coating approach (Fig. 5f). The last layer cures on a substrate which is elastically deformed due to the acceleration field. When the centrifugation is stopped after curing, the substrate relaxes so that the last layer is under compression and the thin film wrinkles^30^.

## Discussion

We have proposed and characterized a passive methodology that freezes an instability at a stage between a compact macroscopic liquid volume and its subsequent dispersion into drops, so as to harness the resulting structure. We have shown that the RTI in polymer films can be rationalized and tamed to tailor the resulting structures. The final structure is elastic, albeit its morphology is underpinned by a combination of fluidic processes: first an instability and then a quasi-static equilibrium. Timing and initial conditions allowed us to control the drop amplitudes, and seeds fostered uniform lattices. While centimeter drops are the norm, we have demonstrated that much smaller scales are attainable, so that this method could be used in the micro-fabrication of soft materials with applications to optics or acoustics, or to control surface properties such as adhesion and wetting. Our method furthers our capacities in fast-prototyping complementing additive manufacturing and other conventional molding techniques. We expect that our approach can be extended to other liquids, which react or undergo a phase transition (other polymers, inks, wax, molten glass, and metals) and other instabilities, therefore opening the way to fabricate a broader repertoire of shapes.

## Methods

### Experimental procedure

Vinylpolysiloxane (VPS) or polydimetylsiloxane (PDMS) were used for the layer fabrication and, for both cases, curing was performed at room temperature (20 °C). For PDMS (Sylgard 184, Dow Corning), the base and curing agent were mixed in a weight ratio of 10:1 using a centrifugal mixer (ARE-310, Thinky) for 30 s at 2000 rpm (clockwise), and then for 30 s at 2200 rpm (counterclockwise). The curing process was sped up by adding an accelerator (3-6559, Dow Corning) to the PDMS, with a weight ratio of 5:1 (PDMS-base to cure-accelerator). VPS (Elite Double 32, Zhermack) was mixed with a weight ratio of 1:1 (base to curing agent) using a centrifugal mixer for 10 s at 2000 rpm (clockwise), and then 10 s at 2200 rpm (counterclockwise). A larger gravity field was emulated either in a lathe (radii of gyration *R* = [57,83.8] mm, rotation speeds Ω = [16.5, 21.9, 34.6,68.9, 102.7] rad s^−1^), in a centrifuge (Sorvall RT6000d, *R* = 110 mm, Ω = [104.7, 157.1, 261.8, 314.2, 356.0] rad s^−1^), in a spin-coater (Laurell WS-650 *R* = 63.5 mm, rotation speeds Ω = 80–1200 rad s^−1^) or in a superspeed centrifuge (Sorvall Lynx4000, *R* = 65 mm, rotation speeds Ω = [523.6, 733.0, 1256, 1571, 1885, 2094] rad s^−1^). To trigger the instability, an array of square or hexagonal holes (diameter 2 mm, length scale *λ*) was laser-cut in an acrylic plate (thickness 1.6 mm) glued on the substrate. The VPS and PDMS solutions were then spin-coated (Laurell WS-650) on the acrylic plate to produce uniform layers of initial thickness in the range 150 ≤ *h*[μm] ≤ 590. The topography of the resulting droplets patterns was digitized using a 3D laser scanner (NextEngine) and the data were post-processed using an in-house Matlab code to quantify the lattice order, length scale, and amplitude of the droplets in the lattice. The microscopic droplets were imaged with a Scanning Electron Microscope Philips XL 30 SEM-FEG. The dynamics of the drop growth was measured by mixing a black dye (Silc-Pig, Smooth-on with a dye concentration of 0.1% in mass) in the PDMS suspension prior to curing using the Beer-Lambert law that relates the transmittance to the thickness and dye concentration^31^. The viscosity of the PDMS polymer solution was characterized with a rheometer (AR-G2, TA Instruments) as a function of time and at a constant temperature (20 °C). The shear rate was fixed at $\dot \gamma \sim u/h\sim 0.1$ s^−1^, consistent with the characteristic drainage velocity and film thickness.

## Electronic supplementary material

Description of Additional Supplementary Files

Supplementary Movie 1

Supplementary Movie 2

## Data Availability

The authors declare that the data supporting the findings of this study are available within the paper and its Supplementary Information Files.
