# Supplementary material for: Designing soft materials with interfacial instabilities in liquid films
Source: Nat Commun. 2018 Oct 26;9:4477. doi: 10.1038/s41467-018-06984-7 (PMC6203858; doi:10.1038/s41467-018-06984-7)
Supplement: Supplementary file 1 — Description of Additional Supplementary Files [file 41467_2018_6984_MOESM1_ESM.pdf]

Description of Additional Supplementary Files

Supplementary Movie 1

Description: A hexagonal array of droplets with regular amplitude bulges to form a dimpled sphere under pressure loading, highlighting the reversible and repeatable behavior of the structure. The scale bar is 10 mm.

Supplementary Movie 2

Description: Rayleigh-Taylor mediated hairy surfaces deform under pressure loading. The scale bar is 1 mm.
